# The association of hearing impairment and its severity with physical and mental health among Chinese middle-aged and older adults

**DOI:** 10.1186/s12955-020-01417-w

**Published:** 2020-05-26

**Authors:** Xin Ye, Dawei Zhu, Siyuan Chen, Ping He

**Affiliations:** 1 School of Public Health, Peking University, 38 Xue Yuan Road, Haidian District, Beijing, 100191 China; 2 China Center for Health Development Studies, Peking University, 38 Xue Yuan Road, Haidian District, Beijing, 100191 China

**Keywords:** Hearing impairment, Chronic diseases, Activities of daily living, Depressive symptoms

## Abstract

**Background:**

Hearing impairment is a common chronic condition which can be closely related to people’s health. However, current studies on this topic are quite limited in developing countries, and few with standardized audiometric measurement and multiple health outcomes. Therefore, we aimed to explore the association between hearing impairment and its severity with physical and mental health among Chinese middle-aged and older adults.

**Methods:**

We obtained data from two sources: (1) China Health and Retirement Longitudinal Study (CHARLS) 2011, 2013, and 2015, in which hearing impairment was measured by asking whether participants aged 45 years old had hearing problems; and (2) Hearing Survey 2019, the baseline survey of a randomized controlled trial conducted in Shandong Province of China, including 376 middle-aged and older participants. The severity of hearing impairment was identified by pure tone average of hearing thresholds at 0.5, 1, 2, and 4 kHz.

**Results:**

In CHARLS, 1248 (8.36%) participants suffered from hearing impairment at baseline, and hearing-impaired individuals were more likely to have chronic diseases, impaired activities of daily living (ADLs), impaired instrumental activities of daily living (IADLs) and depressive symptoms. For the 376 hearing-impaired participants in Hearing Survey 2019, 30.32, 38.30 and 31.38% of them had moderate, severe and profound hearing impairment, respectively. As the severity of hearing impairment increased, individuals were likely to have impaired ADLs, impaired IADLs and depressive symptoms.

**Conclusions:**

Hearing impairment and its severity were closely related to multiple physical and mental health outcomes among Chinese middle-aged and older adults. Actions should be taken to prevent and treat hearing impairment, so as to improve people’s health and well-being.

## Introduction

Hearing impairment is one of the most common sensory dysfunctions, which adds to the burden of physical and mental health extensively [1]. According to the Global Burden of Disease (GBD) 2015 study, half a billion people suffered from disabling hearing impairment (pure tone average of 35 dB or worse) worldwide [2]. Hearing impairment often has a slow onset and progressive deterioration, resulting in unrecognized and under-treated health problems [3]. It brings about lower quality of life, including more comorbidity chronic diseases [4], impaired activities of daily living (ADLs) and instrumental activities of daily living (IADLs) [5, 6], as well as adverse mental health conditions such as depression [7, 8]. The mechanism underlying may be that, hearing impairment can hinder information exchange and social participation, which further impairs active body function, increases psychological burden, and affects people’s health [5, 6].

In addition, the risk of hearing impairment rises rapidly with age, so it is becoming an increasingly serious public health concern [9]. More than 90% of hearing impairments in the world are age-related and irreversible [10]. In China, according to the Second National Sample Survey on Disability in 2006, the prevalence rate of hearing disability (moderate hearing impairment and above) was about 11% among the elderly over 60 [11], ranking top among six categories of disability (hearing, visual, speech, physical, intellectual and mental disabilities) [12]. In 2014–2015 a field survey from four typical provinces in China found that, the prevalence rate of hearing loss was nearly two thirds among Chinese adults aged 60 years and older [13]. In addition, a large proportion of people regard hearing impairment as a natural process of aging which can be ignorable, so hearing impairment has not yet received enough attention as it deserves [14]. In this context, it is imperative to conduct a study on the association between hearing impairment and health outcomes.

However, current studies on this topic are very limited and most of them are conducted in developed countries. It has been shown that hearing impairment brings about poor quality of life in community-dwelling older adults [15], presenting in part as poor physical health and psychological health in developed countries like the U.S. [5, 16], Australia [17, 18], Sweden [19], Finland [16], and Japan [20]. People with hearing impairment are more likely to have chronic diseases [21]. They need more assistance in activities of daily living [22] and have more chances to get affective mood disorders [23]. Only one study in Thailand, a middle-income country in Asia, shows that hearing impairment was associated with poor self-assessed health and psychological health among university adults [24]. In addition, most studies mainly focus on single dimension of health, either physical functions [5, 6] or mental diseases [7, 8]. Therefore, it is essential to further investigate the association of hearing impairment and multiple health outcomes.

Based on the nationally representative data from China Health and Retirement Longitudinal Study (CHARLS) 2011, 2013, 2015 and the latest Hearing Survey 2019, our study incorporates both self-reported hearing data and standardized audiometric measurement, aiming to gain a comprehensive understanding of the association between hearing impairment and its severity with multiple health outcomes in Chinese middle-aged and older adults. This study will draw wide public attention to the association of hearing impairment and multiple health outcomes, as well as turning to early prevention and treatment.

## Methods

### Study sample and population

Currently there is no single database available that could provide information on the variables of hearing impairment and its severity in China. Therefore, we combined two data sets: (1) the CHARLS 2011, 2013, 2015 pooled data; (2) the Hearing Survey 2019 data. The data of CHARLS 2011, 2013, 2015 are based on the national level without the severity of hearing impairment, and the Hearing Survey 2019 is only from a city but with the severity of hearing impairment, which is evaluated by objective audiometric measurement.

The first dataset CHARLS is a long-term tracking project, with its baseline survey conducted in 2011 and follow-up surveys in 2013 and 2015, eying on the community-dwelling Chinese adults aged 45 years old or above [25]. CHARLS adopted the method of PPS (Probability Proportional to Size) and sampling from maps in 450 villages/resident committees, 150 counties/districts and 28 provinces [26], and combined detailed socioeconomic data with high-quality health data to address challenges of China’s aging problem. After data pooling, collating and cleaning, we got the nationally representative 20,099 samples from CHARLS pooled data, with 14,930 samples in baseline 2011.

The second data set Hearing Survey 2019 is the baseline survey of a randomized controlled trial in Linyi City, Shandong Province of China to investigate the association between the severity of hearing impairment and health outcomes. We included middle-aged and older adults with moderate hearing impairment and above (4 frequencies (0.5, 1, 2, 4 kHz) > 40 dB), and with no hearing aids use at baseline. Those in the treatment group were prescribed with hearing aids, while those in the control group received no intervention. The primary outcome is changes in their health conditions over a 12-month period. According to the list provided by the hearing center of Linyi Disabled Persons’ Federation, 500 people were randomly selected, of which 36 patients were approached and were unwilling to give informed consent. In addition, 12 patients were excluded from the study because they were unable to understand the questionnaire, and 22 patients did not meet the inclusion criteria for the Hearing Survey 2019. We finally got the sample size of 430, of which 376 people were 45 years old and above.

### Independent variables: hearing impairment and its severity

In CHARLS 2011, 2013 and 2015, hearing status was derived by asking whether respondents have hearing problems or not. The result is a subjective answer “yes” or “no”. Prior studies have proved that a single-item question about an individual’s hearing ability is moderately useful and valid to assess hearing and can be used for a population-based study [27, 28].

Hearing Survey 2019 measures hearing status by pure-tone average (PTA) at the thresholds of 0.5, 1, 2, and 4 k Hz. According to the classification by World Health Organization, those are defined as moderate hearing impairment if PTA > 40 dB and PTA ≤ 60 dB, severe hearing impairment if PTA > 60 dB and PTA ≤ 80 dB, and profound hearing impairment if PTA > 80 dB [29].

### Outcome variables: physical and mental health status

In both CHARLS and Hearing Survey, we incorporated several variables to measure health status. Physical health was measured by whether they had chronic diseases [30], impaired ADLs [31] and impaired IADLs [32]; Mental health was measured by whether they had depressive symptoms [33].

1. Chronic diseases: Individuals were asked whether they had been diagnosed with chronic diseases, including hypertension, dyslipidemia, diabetes, cancer, chronic lung disease, liver disease, heart attack, stroke, kidney disease, digestive disease, arthritis or asthma. If individuals reported having at least one of these chronic diseases, we defined them as having chronic diseases.
2. ADLs: Individuals were asked whether they had any difficulty with activities of daily living, including dressing, bathing or showering, eating, getting into or out of bed, using the toilet, or controlling urination and defecation. Impaired ADLs was defined as difficulty or inability with any of the activity [5].
3. IADLs: Individuals were asked whether they had any difficulty with instrumental activities of daily living, including doing household chores, preparing hot meals, shopping for groceries, making phone calls, taking medications, or managing money. Impaired IADLs was defined as difficulty or inability with any of the activity [5].
4. Depressive symptoms: Both surveys applied the screening tool Center for Epidemiologic Studies Depression Scale-10 items (CES-D-10) Chinese version to detect depressive symptoms [34]. The CES-D-10 Chinese version has been shown to have good sensitivity, specificity, and predictive value [35]. Subjects responded to the CES-D by rating the frequency of each mood occurred during the past week on a four-point scale, ranging from 0 (“none of the time”) to three (“most of the time”) [34]. A cut-off score ≥ 10 on the total 0–30 CES-D-10 was optimal to identify individuals at risk of depressive symptoms [36].

### Covariates

Prior research has identified several confounders that are associated with hearing impairment and health, and should be included in the analysis as covariates [5, 7, 18]. These include age (continuous variable), gender (male or female), residency (rural or urban), educational attainment (illiterate, finishing primary school, finishing middle school and above), marital status (married or partnered; alone) and annual income. However, income is a less useful measure in late life due to exiting the labor force [37], and its missing value occupies nearly half in CHARLS, so we decided not to include income as the covariate.

### Statistical analyses

Descriptive analyses were used to present sample characteristics and hearing status. We used analysis of Variance (ANOVA) and the Chi-square test to compare characteristics between different hearing impairment categories. Logistic regression models and marginal effects were employed to predict the probabilities of having chronic diseases, impaired ADLs, impaired IADLs, and depressive symptoms with changes in hearing status while potential confounders were controlled [38]. Before conducting the logistic regression, we have tested the dual collinearity of independent variables by the correlation matrix and the variance inflation factor (VIF) [39], and found no strong dual collinearity of independent variables in the Logistic regression. The software Stata version 14.0 for Mac was utilized for statistical analyses. All hypothesis tests were two-sided, with a *P*-value less than 0.05 considered statistically significant.

## Results

### Participants’ characteristics

The characteristics of CHARLS Baseline 2011 are shown in Table 1. The mean age of participants was 58.87 years old, with nearly half being male (48.06%), illiterate (45.47%), most residing in rural areas (77.68%) and being married (89.60%). There were 1248 (8.36%) participants suffering from hearing impairment. Compared to those with no hearing impairment, hearing-impaired participants tended to be older, male, less educated, residing in rural areas, being alone and were more likely to have chronic diseases, impaired ADLs, impaired IADLs, and depressive symptoms. Table 1 shows more detailed features for participants in CHARLS Baseline 2011.

**Table 1:** Descriptive Characteristics of Study Participants from Baseline China Health and Retirement Longitudinal Study 2011 (*N* = 14,930)

Characteristic	All		No Hearing Impairment		Hearing Impairment		***p*** Value
	n	%	n	%	n	%	
N	14,930	100.00	13,682	91.64	1248	8.36	
Age (S.D.)	58.87 (0.08)		58.29 (0.08)		65.20 (0.29)		< 0.001
Gender							0.006
Male	7175	48.06	6529	47.72	646	51.76	
Female	7755	51.94	7153	52.28	602	48.24	
Education							< 0.001
Illiterate	6789	45.47	6006	43.90	783	62.74	
Primary School	3177	21.28	2936	21.46	241	19.31	
Middle School and above	4964	33.25	4740	34.64	224	17.95	
Residency							< 0.001
Urban	3332	22.32	3131	22.88	201	16.11	
Rural	11,598	77.68	10,551	77.12	1047	83.89	
Marriage							< 0.001
Married	13,377	89.60	12,375	90.45	1002	80.29	
Alone	1553	10.40	1307	9.55	246	19.71	
Chronic Diseases							< 0.001
0	4542	30.42	4270	31.21	272	21.79	
≥ 1	10,388	69.58	9412	68.79	976	78.21	
ADLs							< 0.001
No-impaired	12,478	83.58	11,625	84.97	853	68.35	
Impaired	2452	16.42	2057	15.03	395	31.65	
IADLs							< 0.001
No-impaired	11,802	79.05	11,087	81.03	715	57.29	
Impaired	3128	20.95	2595	18.97	533	42.71	
Depressive Symptoms							< 0.001
No	9408	63.01	8816	64.44	592	47.44	
Yes	5522	36.99	4866	35.56	656	52.56	

*ADLs* Activities of daily living, *IADLs* Instrumental activities of daily living

The characteristics of Hearing Survey 2019 for 376 participants aged 45 years old and above are shown in Table 2. The mean age of participants was 68.31 years, with 70.21% male, 46.54% illiterate, 92.29% residing in rural areas and 77.39% being married. In terms of hearing impairment severity, 114 (30.32%) participants have moderate hearing impairment, 144 (38.30%) with severe hearing impairment, and 118 (31.38%) with profound hearing impairment. Participants with more severe hearing impairment tend to be with impaired IADLs and depressive symptoms. Table 2 shows more detailed features for participants in Hearing Survey 2019.

**Table 2:** Descriptive Characteristics of Study Participants from Hearing Survey 2019 (*N* = 376)

Characteristic	All		Moderate Hearing Impairment		Severe Hearing Impairment		Profound Hearing Impairment		***p*** Value
	n	%	n	%	n	%	n	%	
N	376	100.00	114	30.32	144	38.30	118	31.38	
Age (S.D.)	68.31(0.53)		68.68 (0.97)		69.50 (0.85)		66.52 (0.92)		0.057
Gender									0.644
Male	264	70.21	82	71.93	103	71.53	79	66.95	
Female	112	29.79	32	28.07	41	28.47	39	33.05	
Education									0.108
Illiterate	175	46.54	52	45.61	66	45.83	57	48.30	
Primary School	127	33.78	31	27.19	56	38.89	40	33.90	
Middle School and above	74	19.68	31	27.19	22	15.28	21	17.80	
Residency									0.895
Urban	29	7.71	9	7.89	10	6.94	10	8.47	
Rural	347	92.29	105	92.11	134	93.06	108	91.53	
Marriage									0.240
Married	291	77.39	94	82.46	106	73.61	91	77.12	
Alone	85	22.61	20	17.54	38	26.39	27	22.88	
Chronic Diseases									0.875
0	109	28.99	35	30.70	40	27.78	34	28.81	
≥ 1	267	71.01	79	69.30	104	72.22	84	71.19	
ADLs									0.053
No-impaired	290	77.13	97	85.09	106	73.61	87	73.73	
Impaired	86	22.87	17	14.91	38	26.39	31	26.27	
IADLs									0.003
No-impaired	121	32.18	49	42.98	46	31.94	26	22.03	
Impaired	255	67.82	65	57.02	98	68.06	92	77.97	
Depressive Symptoms									0.001
No	222	59.04	79	69.30	89	61.81	54	45.76	
Yes	154	40.96	35	30.70	55	38.19	64	54.24	

*ADLs* Activities of daily living, *IADLs* Instrumental activities of daily living

### The association between hearing impairment and multiple health outcomes

Table 3 shows the association of hearing impairment and its severity with the prevalence and odd ratio of having chronic diseases, impaired ADLs, impaired IADLs and depressive symptoms. There were 69–82% of the respondents having chronic diseases, 14–27% reporting impaired ADLs, 21–78% reporting impaired IADLs and 31–53% having depressive symptoms.

**Table 3:** Prevalence and Odds Ratio of Chronic Diseases, Impaired Activities of Daily Living, Instrumental Activities of Daily Living, and Depressive Symptoms by Hearing Status

Hearing Status	Chronic Diseases		Impaired ADLs		Impaired IADLs		Depressive Symptoms	
	Prevalence (95%CI)	OR (95%CI)	Prevalence (95%CI)	OR (95%CI)	Prevalence (95%CI)	OR (95%CI)	Prevalence (95%CI)	OR (95%CI)
CHARLS Pooled Data (*N* = 14,930)								
Hearing Status								
No Hearing Impairment (Reference Group)	0.74 (0.73–0.74)	1.00	0.17 (0.16–0.18)	1.00	0.21 (0.20–0.22)	1.00	0.33 (0.32–0.34)	1.00
Having Hearing Impairment	0.82 (0.81–0.83)	1.60^***^ (1.49–1.72)	0.27 (0.26–0.28)	1.86^***^ (1.75–1.98)	0.36 (0.35–0.37)	2.26^***^ (2.13–2.39)	0.43 (0.41–0.44)	1.57^***^ (1.48–1.67)
Hearing Survey Data (*N* = 376)								
Hearing Status								
Moderate Hearing Impairment (Reference Group)	0.69 (0.61–0.78)	1.00	0.14 (0.08–0.21)	1.00	0.57 (0.48–0.67)	1.00	0.31 (0.23–0.40)	1.00
Severe Hearing Impairment	0.71 (0.64–0.79)	1.10 (0.63–1.93)	0.26 (0.19–0.33)	2.14^*^ (1.12–4.10)	0.67 (0.60–0.75)	1.53 (0.91–2.59)	0.39 (0.31–0.47)	1.41 (0.82–2.42)
Profound Hearing Impairment	0.72 (0.65–0.80)	1.17 (0.65–2.10)	0.27 (0.19–0.35)	2.26 ^*^ (1.15–4.44)	0.78 (0.71–0.86)	2.76^***^ (1.53–4.97)	0.53 (0.44–0.62)	2.56^***^ (1.47–4.45)

Component scores are adjusted for age, gender, education, residency and marriage. The means are given first, followed parenthetically by confidence intervals. *ADLs* Activities of daily living, *IADLs* Instrumental activities of daily living, *OR* Odd Ratios, *CI* Confidence Intervals

^*^P < 0.05,^**^P < 0.01,^***^P < 0.001

Results from CHARLS pooled data indicated that, hearing impairment was significantly related to the greater risk in physical and mental health. Compared with those having no hearing impairment, the prevalence of having chronic diseases (OR = 1.60, 95% CI = 1.49–1.72), impaired ADLs (OR = 1.86, 95% CI = 1.75–1.98), impaired IADLs (OR = 2.26, 95% CI = 2.13–2.39) and depressive symptoms (OR = 1.57, 95% CI = 1.48–1.67) increased if individuals had self-reported hearing impairment.

### The association between the severity of hearing impairment and multiple health outcomes

Results from Hearing Survey 2019 showed that, different levels of hearing impairment severity were linked to different risks of having impaired ADLs, IADLs, and depressive symptoms.

To be exact, for those with severe and profound hearing impairment, the prevalence of impaired ADLs was significantly higher than the reference group (people with moderate hearing impairment) (OR = 2.14, 95% CI = 1.12–4.10; OR = 2.26, 95% CI = 1.15–4.44). For those with profound hearing impairment, the prevalence of impaired IADLs (OR = 2.76, 95% CI = 1.53–4.97) and depressive symptoms (OR = 2.56, 95% CI = 1.47–4.45) was significantly higher than the group of moderate hearing impairment.

Among people with moderate to profound hearing impairment, the prevalence of chronic diseases did not show large differences compared with moderately hearing-impaired people (OR = 1.10, 95% CI = 0.63–1.93; OR = 1.17, 95% CI = 0.65–2.10). And groups with moderate and severe hearing impairments show no significant differences in the prevalence of having impaired IADLs (OR = 1.53, 95% CI = 0.91–2.59) and depressive symptoms (OR = 1.41, 95% CI = 0.82–2.42).

## Discussion

With the population aging, an increasing number of people are living with hearing impairment, especially during their later-life years, which can be associated with multiple health problems. Based on two data sets from nationally representative CHARLS pooled data and the latest Hearing Survey 2019 data, this study is the first to present the association of hearing and its severity with multiple health outcomes in China. Overall, hearing-impaired individuals in CHARLS were more likely to have chronic diseases, impaired ADLs, impaired IADLs and depressive symptoms. For the hearing-impaired participants in Hearing Survey 2019, as the severity of hearing impairment increases, individuals were also more likely to have impaired ADLs, impaired IADLs and depressive symptoms.

What we have found among hearing-impaired adults in China is generally consistent with previous studies about the effects of hearing impairment on people’s physical and mental health. People with more severe hearing impairment tend to be older [9], male [40], and less educated [41]. And hearing impairment has been proved to correlate with more chronic diseases [21]. With the deterioration of hearing impairment, people are more likely to have poorer body functions measured by activities of daily living (ADLs) and instrumental activities of daily living (IADLs) [5, 6]. In addition, hearing impairment can also lead to depressive symptoms, such as sadness, hopelessness, helplessness [42], and exacerbating the decline in individuals’ psychosocial well-being [43].

Our results further demonstrated the association of different severity of hearing impairment with physical and mental health outcomes. The prevalence of impaired ADLs showed an elevated increase for those with severe to profound hearing impairment, compared with moderate hearing impairment. For those with profound hearing impairment, the prevalence of impaired IADLs and depressive symptoms were much higher than those with moderate hearing impairment. The underlying mechanism may be that, since ADLs measures basic functions in daily activities and IADLs measures functions in more subtle activities, hearing impairment may be more related to the decline in certain brain structures that control these functions [5]. And hearing impairment inevitably brings about communication barriers, thus the more probability of falling into depression is conceivable [42]. However, the prevalence of chronic diseases did not show significant differences within groups of severe to profound hearing impairment, which may due to the limited sample size or the selection of reference group.

Since hearing impairment can be difficult to detect and cure, and is closely related to health, it is imperative to raise awareness and take effective measures. For newborns, the screening and prevention of hearing impairment should be taken as early as possible. For those with hearing impairment, especially with moderate and above hearing impairment, it is necessary to provide rehabilitative devices to compensate for the loss of functions, such as wearing hearing aids [44], as people who use hearing aids manifested better self-care, lower levels of depression, and better overall health [45]. However, due to the high price of hearing aids, the accessibility and utilization rate are relatively low worldwide [46]. Our findings highlight the need to improve the worse health of hearing-impaired population.

Limitations of our study are that, first, the heterogeneity between two different data sets should be noted, and extrapolation from the Hearing Survey 2019 should be cautious because the sample is from only one city, which may not be representative of the hearing-impaired adults in China. Second, some of the hearing and health variables are determined by participants’ self-reported data, which may bring some bias. Third, although some confounders have been controlled, other undetectable confounders may also affect the results [5].

Despite of above limitations, the main strength lies in that, our study focuses on the association between hearing impairment and health outcomes in China, which is a relatively unexplored and prospective topic in developing countries. To be exact, we not only used 3 waves of national representative CHARLS data, but took a step further to investigate different severity of hearing impairment by the latest Hearing Survey 2019 to elucidate the complex interplay between hearing impairment and health. We applied multiple physical and mental health outcomes to demonstrate the association from different perspectives and we combined both self-reported hearing status and objective audiometric measurement, all of which can act as more profound and compelling evidence to the extant literature.

## Conclusions

The high prevalence and increasing severity of hearing impairment have been a tendency in the aging society. Hearing impairment and its severity were found to be closely correlated with physical and mental health in Chinese middle-aged and older adults. Immediate actions should be taken to prevent and treat hearing impairment, so as to improve their health and well-being of hearing-impaired people.

## Data Availability

The data supporting the conclusion of this article are includes within the article. Any queries regarding these data may be directed to the corresponding author.

## Abbreviations

CHARLS: China Health and Retirement Longitudinal Study
ADLs: Activities of daily living
IADLs: Instrumental activities of daily living
OR: Odd Ratios
CI: Confidence Intervals
ANOVA: Analysis of variance
